# A Study of Clinical Profile and Treatment in Adult Hemophilia Patients with Special Reference to the Inhibitor Levels

**DOI:** 10.7759/cureus.54663

**Published:** 2024-02-21

**Authors:** Renuka Vasava, Minal Shastri, Vaishnavi M Rathod, Gayatri Laha, Vaishnovi Vaishnovi, Nipakumari J Patel, Rajani Deshagoni, Prerna Singh, Nandan Joshi, Darshankumar M Raval

**Affiliations:** 1 General Medicine, Sir Sayajirao General Hospital, Medical College Baroda, Vadodara, IND; 2 General Medicine, Parul Institute of Medical Sciences and Research, Vadodara, IND; 3 Internal Medicine, Soochow University, Suzhou, CHN; 4 Internal Medicine, Rajiv Gandhi Institute of Medical Sciences, Adilabad, IND; 5 Infectious Disease Department, Mayo Clinic, Jacksonville, USA; 6 Internal Medicine, Surat Municipal Institute of Medical Education and Research, Surat, IND

**Keywords:** hemophilia in adult, severity of hemophilia, treatment, factor replacement, factor viii inhibitors, factor levels

## Abstract

Introduction

Hemophilia is an uncommon, X-linked recessive bleeding condition characterized by a lack of either factor VIII or factor IX. It is more prevalent in men. Due to the substantial impact inhibitor development has on patient prognosis, the primary treatment for hemophilia is the transfusion of recombinant factors. The aim of our study is to investigate 40 adult patients with hemophilia in terms of their clinical profile, clinically relevant risk factors for inhibitor development, therapy-related aspects such as treatment duration, factor requirements, transfusion frequency, presence of inhibitors, and complications.

Methods

This cross-sectional observational study involving 40 patients of hemophilia over 12 years of age was conducted at a tertiary care hospital in Gujarat. Data on sociodemographic characteristics, presenting complaints, bleeding episodes, hemophilia type, and medical history were gathered over a one-year span. Patients were stratified into mild, moderate, and severe groups based on their respective levels of factor activity. Various parameters, including the frequency of factor therapy, percentage of factor concentrate, inhibitor presence, and disease and therapy-related complications, were analyzed. The distribution of patients across these parameters was calculated and illustrated using pie charts.

Results

Nineteen out of 40 patients were from 20 to 40 years of age. The majority of cases (n=24), however, had been diagnosed before the patients reached the age of 10. All patients were male, and half of the patients (n=20) suffered from mild disease. The most common site of bleeding was the knee joint, and 33 cases had one to 10 bleeding episodes per year. Thirty-two out of 40 patients needed less than 40 factor vial transfusions, whereas eight needed more than 40 factor vial transfusions. Two cases of severe disease were positive for inhibitors of factor VIII, whereas one patient was found to have a hepatitis B virus (HBV) infection.

Conclusions

Hemophilia, a rare bleeding disorder, has primarily been studied in pediatric populations. This study, however, shifts the focus toward adult individuals. Our cohort consisted exclusively of male patients, with the predominant group diagnosed with hemophilia A and falling within the age range of 20 to 40 years. Most patients had been diagnosed before 10 years of age. The primary complication observed was joint bleeding, with the knee joint being the most commonly affected site. Approximately two-thirds of cases had a history of minor trauma necessitating factor replacement, yet only 5% exhibited the presence of inhibitors.

## Introduction

Hemophilia represents a genetic anomaly within the blood coagulation system characterized by a deficiency in either factor VIII or factor IX. Hemophilia A and B are the two primary types of this condition, each resulting from single-gene mutations that affect the coding for factors VIII and IX, respectively. Globally, bleeding disorders manifest in approximately one in every 1000 individuals, with hemophilia A constituting 70% of cases [1]. In India, the prevalence rate is 0.7 per lakh, totaling an estimated 54,454 diagnosed cases. The prevalence rate of hemophilia B in India is 0.1 per 100,000, a figure 13 times lower than the 1.3 per 100,000 prevalence rate in the United States (US) [1]. A notable concern in India is the underdiagnosis of the condition, in stark contrast to the US, which benefits from a more comprehensive surveillance system [1].

The inheritance pattern of hemophilia is X-linked recessive, affecting males who receive a single X chromosome from their mothers. This genetic transfer gives males a 50% probability of inheriting the disorder if the mother is an asymptomatic carrier. Females, on the other hand, must inherit defective alleles from both parents to manifest the disease, owing to the X-linked recessive inheritance pattern. Consequently, males exhibit clinical symptoms of the disorder, whereas female carriers of the mutated gene generally remain asymptomatic. It is important to note that approximately 80% of affected females possess a de novo mutation, and in 30% of hemophilia cases, there is an absence of a family history of the disease.

Due to the deficiency of clotting factors VIII and IX in hemophilia, patients are predisposed to an increased risk of bleeding or thrombosis, given the pivotal role these factors play in the intrinsic and common coagulation pathways. From a clinical perspective, this disorder is divided into three severity levels: mild, moderate, and severe, determined by the activity level of the clotting factor. Notably, severe bleeding episodes may manifest in various sites, including the genitourinary system, mucosal membranes, gums, and particularly the joints, with the knee joints being especially susceptible. Additionally, instances of severe bleeding in critical regions, such as the brain, gastrointestinal tract, and neck, have been documented, each presenting a substantial risk to patient survival [2].

The cornerstone of hemophilia treatment is factor replacement therapy, which utilizes either plasma-derived products or factor VIII concentrates to restore hemostasis. However, the efficacy of this treatment is constrained by the risks of transfusion-related viral transmission in plasma-derived products and the development of inhibitors in patients undergoing pure recombinant factor VIII and IX replacement therapy. The formation of inhibitory antibodies against recombinant factor VIII is particularly problematic, with up to 25% of patients with severe hemophilia A developing factor VIII inhibitors [3]. Factors contributing to the development of these inhibitors encompass genetic mutations (within the major histocompatibility complex and factor VIII genes), immunogenic responses (linked to concurrent infections and frequent transfusions), environmental factors (such as the age at which replacement therapy commences), and the overall severity of the disease. The occurrence of inhibitors significantly complicates the management of acute bleeding episodes, as evidenced by refractory bleeding, which typically signals the presence of these antibodies. Consequently, addressing the challenge of inhibitors including their treatment, prediction, and prevention post-exposure to factor VIII has emerged as a critical focus in the management of hemophilia.

This study is designed to delineate the clinical profile of adult patients with hemophilia and scrutinize therapy-related factors, including the duration and frequency of treatment, factor requirements, and any complications arising from therapy. Furthermore, it aims to estimate the levels of inhibitors against factor concentrates and identify clinically relevant risk factors for the development of inhibitors within the therapeutic context.

## Materials and methods

Following receipt of approval and clearance from the scientific review committee and institutional ethics committee for human research (approval number: IECHR-PGR/14-19), a population-based, observational cross-sectional study was conducted on 40 adult hemophilia patients at Shri Sayajirao Gaekwad General (SSG) Hospital, Vadodara, Gujarat, spanning from January 2019 to January 2020.

This research encompassed all hemophilia A and B patients aged 12 years and above, comprising individuals admitted to hospital wards, attending follow-up clinics for replacement therapy, and those enrolled from the community registered in the Hemophilia Society Chapter One Registry.

After explaining the study's objectives and securing written informed consent from the participants, data were collected utilizing a structured questionnaire. This instrument gathered information on sociodemographic variables (age, gender), presenting complaints, frequency of bleeding episodes (categorized as fewer than 10, 10-15, or more than 15 episodes per year), age at initial diagnosis, clinical manifestations, type of hemophilia (A or B), and past medical history (e.g., blood transfusions, complications). A positive family history was acknowledged if the patient's father or male relatives on the maternal side were previously diagnosed with hemophilia.

Normal levels for factors VIII and IX are established to be within the 50-150% range. Patients were accordingly classified into different severity categories based on these factor levels in the blood: mild for factor levels above 5% and below 40%, moderate for levels between 1% and 5%, and severe for levels under 1% [4].

The primary treatment strategy for hemophilia patients at SSG Hospital consists of factor replacement therapy, employing recombinant factor VIII or IX concentrate. Additionally, transfusions of blood products, including packed cell volume (PCV), fresh frozen plasma (FFP), and cryoprecipitate, are utilized to address anemia and manage bleeding, particularly in instances awaiting factor availability. Based on the estimated annual requirement of 20,000 IU of clotting factor VIII per patient, participants were divided into two groups according to the annual number of vials used per patient [1]. A threshold of 40 vials per year was established (one vial equals 500 IU of factor VIII).

The study focused on various factors associated with factor therapy in hemophilia patients, including the total dosage necessary to achieve hemostasis, its administration frequency as indicated by the annual vial usage, and the varying dosage requirements based on the severity of hemophilia. The investigation also encompassed the assessment of the percentage of factor concentrate and inhibitors, as well as the evaluation of hemophilia-related complications such as arthropathy, gastrointestinal bleeding, and intracranial bleeding. Therapy-related complications, like transfusion-related infections, were also explored.

Annual assessments of inhibitor levels, undertaken by a local hemophilia society for each participant, were integral to the study. Notably, only the most recent evaluations, gathered within the preceding 12 months, were incorporated into our dataset for subsequent analysis.

Following the collection of data, the results were presented in diverse chart and table formats, allowing for a clear and accessible representation of the research outcomes. The selection of visual aids-including bar graphs, pie charts, histograms, and tables-was strategically chosen to effectively convey the diverse dimensions of the study's results.

## Results

**Figure 1:**
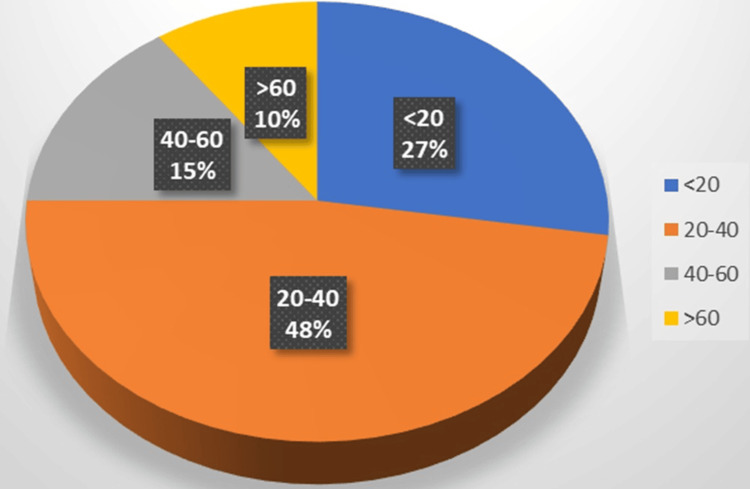
Patient proportion according to age groups Proportion of patients according to age groups (in years) <20 years - 27% (n=11), 20-40 years - 48% (n=19), 40-60 years - 15% (n=6), >60 years - 10% (n=4)

In our cohort of 40 patients, 19 were aged between 20 and 40 years, representing the largest age group. Conversely, the smallest group comprised individuals aged 60 years and above, totaling only four patients (Figure 1).

Notably, a significant majority (60%, n = 24) were diagnosed before the age of 10. Diagnoses were made between the ages of 10 and 20 years in approximately 25% of cases (n = 10), and 15% (n = 6) were diagnosed after the age of 20 years. Importantly, data pertaining to pediatric patients (under 12 years) were excluded from this analysis.

The study exclusively involved male participants, with no female cases of hemophilia identified within our sample. Hemophilia A was overwhelmingly prevalent, accounting for 97.5% of the group (n = 39), while hemophilia B was rare, observed in only 2.5% (n = 1) of the cases. Despite the genetic nature of hemophilia, a family history of the disorder was reported in only half of the cases, with the remainder lacking any known family history of hemophilia. The severity classification was based on factor VIII or IX levels. In the study population, 50% of cases exhibited mild hemophilia, while 25% were categorized as having moderate and severe hemophilia, respectively.

Joint bleeding was the most frequent presentation, with 60% of patients (n = 24) experiencing bleeding into the knee joint. This was followed by the elbow joint (22.5%, n = 9), gum bleeding (20%, n = 8), and the ankle joint (10%, n = 4). The hip joint was least affected, as noted in only one case (2.5%).

**Figure 2:**
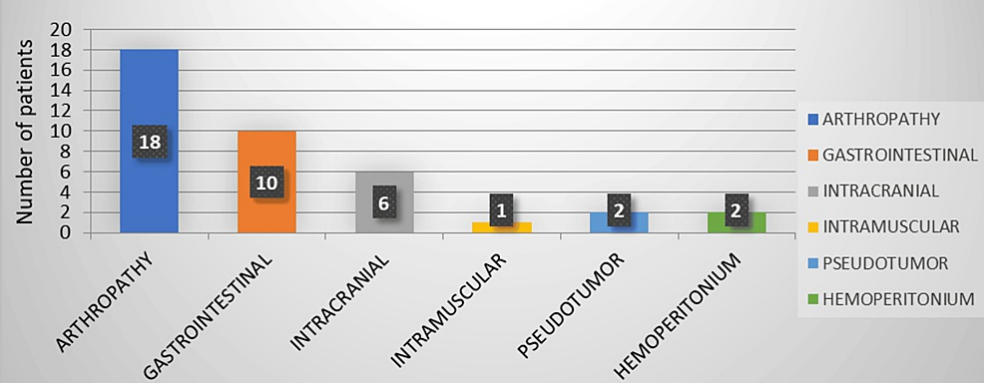
Complications (In number of patients affected)

Arthropathy emerged as the most common complication, affecting nearly half of the patients (Figure 2).

Gastrointestinal bleeding was detailed as gum bleeding (20%, n = 8), hematemesis (2.5%, n = 1), and bleeding per rectum (2.5%, n = 1). The majority of patients (82.5%, n = 33) experienced 1-10 bleeding episodes annually, predominantly mild episodes not necessitating factor replacement therapy. A small fraction (5%, n = 2) reported more than 15 bleeding episodes per year. Among the cohort, 80% (n = 32) required fewer than 40 vial transfusions (equivalent to 20,000 IU) of clotting factors annually, while 20% (n = 8) needed more than 40 vial transfusions. Nonetheless, given the limited sample size, no significant correlation could be drawn between the number of vials transfused and disease severity.

Approximately 70% of the cohort (n = 28) reported incidents of minor trauma necessitating factor replacement therapy. In cases of mild hemophilia, the minimum dosage of factor VIII required to achieve hemostasis was identified as 700 IU. This requirement increased to 2000 IU for moderate hemophilia, with severe cases demonstrating a significantly higher maximum dosage need in comparison to mild and moderate conditions (Table 1).

**Table 1:** Total dosage of factor VIII required to achieve hemostasis according to severity of hemophilia

Severity	No. of Patients	Dosage of Factor VIII	(IU)
		Minimum	Maximum
Mild	20	700	62,000
Moderate	10	2000	60,000
Severe	10	2000	1,35,000

Within the study population, only two individuals (5%) exhibited positive tests for inhibitors against factor VIII, all of whom were classified within the severe hemophilia category, marked by factor VIII levels below 1%.

In the context of transfusions and related complications, half of the participants acknowledged a history of receiving blood product transfusions (PCV, FFP, and cryoprecipitate). Notably, among the 40 patients with hemophilia, only a single case (2.5%) was diagnosed with a hepatitis B virus (HBV) infection.

## Discussion

Type A, commonly referred to as classic hemophilia, is characterized by a deficiency in factor VIII, a protein crucial for blood clot formation. Hemophilia B, on the other hand, results from a deficiency in factor IX. Severe manifestations of the disease are observed in 45% of individuals with hemophilia A and 35% with hemophilia B [5]. According to Castaman and Matino, the gene responsible for factor VIII is located at the extremity of the X chromosome's long arm, whereas the gene for factor IX lies proximal to the centromere of the long arm. The factor VIII gene, characterized by its considerable size and complex architecture, comprises 26 exons, in contrast to the significantly smaller factor IX gene, which includes only eight exons. Variabilities such as insertions, deletions, point mutations, and rearrangements have been documented in both genes, albeit at differing frequencies. Major genetic mutations contribute to approximately 7% of hemophilia B cases. Conversely, gene rearrangements, particularly intron 22 inversion, are prevalent in nearly half of the severe hemophilia A cases [6].

Within our research cohort, with the exception of one patient diagnosed with hemophilia B, nearly all cases (97.5%) were attributed to hemophilia A. This predominance aligns with findings from Mishra et al. and Nigam et al., who reported hemophilia A prevalence rates of 88.3% (68 out of 77) and 88.1% (224 out of 254) within their cohorts, respectively [7,8].

According to the Indiana Hemophilia & Thrombosis Center's report, approximately 25% of individuals with hemophilia experience a mild variant of the condition, with 15% exhibiting moderate symptoms and a significant 60% enduring severe manifestations [4]. In contrast, our study presented a different distribution among evaluated patients: mild hemophilia was observed in 50% of patients, moderate in 25%, and severe in the remaining 25%. This diverges notably from Mishra et al., who observed a predominance of severe disease (80.5%) and mild to moderate forms in fewer than 20% of their study group [7]. However, Nigam et al.'s findings revealed no marked disparity in disease severity across categories, with their patient distribution nearly equal at 32.28% mild, 37.4% moderate, and 30.32% severe [8].

Hemophilia A arises from mutations in the F8 gene on the X chromosome. Typically, females possess two X chromosomes, while males have one X and one Y chromosome. This genetic arrangement makes males more vulnerable to X-linked disorders such as hemophilia A, as they lack a second X chromosome that could potentially offer a compensatory function for the mutated gene, as seen in females [9]. Females generally act as asymptomatic carriers, with the manifestation of the condition in females being exceptionally rare. This was evident in our study, which comprised solely male patients.

Individuals with severe hemophilia A are often diagnosed within the first two years of life, often identified following episodes of oral or soft tissue bleeding, procedural interventions, or through a familial history of the condition. Conversely, those with mild hemophilia A are typically diagnosed later in life [10]. In our study, 15% of cases diagnosed with mild hemophilia were identified after the age of 20, aligning with the findings of Nigam et al., who noted a comparable proportion of 17% diagnosed beyond this age [8].

Approximately 70% of hemophilia cases report a familial history of the disorder, with the remaining 30% lacking any identifiable familial link, potentially due to spontaneous genetic mutations [4]. In our study, 50% of participants (20 out of 40 cases) had a documented positive family history, closely mirroring the prevalence reported by Mishra et al., where 52.2% of patients demonstrated a family history of hemophilia [7].

Clinically, hemophilia is characterized by bleeding episodes following minor trauma or occurring spontaneously, with the severity often correlated with residual factor levels. In cases of mild hemophilia, bleeding is predominantly noted after significant surgery or trauma, while spontaneous episodes are seldom observed. Moderate hemophilia manifests through bleeding following injury, trauma, surgical procedures, or dental work, with up to 25% of patients experiencing recurrent joint bleeds. Conversely, severe hemophilia is frequently associated with spontaneous bleeding episodes [11]. Our analysis revealed joint bleeding as the primary symptom (45%, n = 18), reflecting the findings of Rajendra et al., where 64.96% of patients reported joint hemorrhages. Gum bleeding was identified as the second most common symptom in both cohorts, constituting 20% of our study group and 7.08% in Nigam et al.'s research [8].

According to the World Federation of Hemophilia, the knees are the most frequently impacted joints (45%), followed by elbows (30%), ankles (15%), shoulders (3%), and wrists (2%) [12]. Similarly, our study revealed that weight-bearing joints, notably knees (60%) and ankles (10%), predominantly experienced hemarthroses. These findings align with Mishra et al.'s research, which also noted knee joint involvement in approximately 57.1% of cases [7].

Nigam et al. observed that trauma-induced bleeding accounted for 43.30% (110 out of 254) of episodes, with the majority of patients (56.69%, 144 out of 254) experiencing spontaneous bleeding necessitating factor replacement therapy [8]. In contrast, our analysis identified that a substantial majority, 70% (28 out of 40), had histories of minor trauma leading to the need for factor replacement.

In our cohort, HBV infection was detected in one individual (2.5%) out of 40 cases. Comparatively, Mishra et al. noted hepatitis B and C infections in 6.5% and 9.1% of their patients, respectively [7]. Dubey et al. reported seropositivity rates for transfusion-related infections among hemophilia patients at 1.75% for HIV, 1.75% for HBsAg, and 13.15% for HCV [13].

Moreover, 5% of our patients (two out of 40) were found to have inhibitors, all of whom had severe hemophilia with factor VIII levels below 1%. This finding is in close agreement with the study by Dubey et al., where the overall prevalence of inhibitors in hemophilia A patients was 5.1% [13]. However, Ghosh et al. documented the presence of inhibitors in 8% of 292 severe and 5.5% of 36 moderate hemophilia A patients [14]. The relatively low incidence of inhibitors observed in our patients, a trend also mirrored in developing countries such as India, may be attributed to the delayed initiation of factor replacement therapy and the limited or inconsistent availability of pure factor concentrates [13]. These observations align with the findings of the CANAL trial (A Study of Nalbuphine Extended Release in Idiopathic Pulmonary Fibrosis for Treatment of Cough), which demonstrated that the intensity of therapy is directly associated with an increased incidence of inhibitors, surpassing several other investigated risk factors [15].

Limitations

The study's generalizability is constrained by its modest cohort size, which was predominantly sourced from a single center in Baroda. Consequently, the applicability of the findings to broader and more heterogeneous populations may be limited. Furthermore, being a cross-sectional observational study, it did not delve into specific risk factors associated with the emergence of inhibitors. The majority of participants were diagnosed with mild hemophilia, correlating with a lower frequency of factor transfusion. Furthermore, the identification of inhibitors against factor concentrates in only two patients restricted the capacity for a detailed analysis.

## Conclusions

Hemophilia, an inherited bleeding disorder, predominantly affects males, attributable to its X-linked recessive transmission. Within the cohort of 40 male patients in our study, only half reported a family history of hemophilia. Notably, hemophilia A was the predominant condition in our sample, with a single instance of hemophilia B. Approximately half of the participants were aged between 20 and 40 years; however, the majority were diagnosed with hemophilia before reaching 10 years of age. Joint bleeding, especially in the knee joints, emerged as the most frequent initial symptom, followed by gum bleeding. The data revealed that the majority of patients exhibited a mild form of the condition. The foundation of hemophilia management in this cohort was factor replacement therapy, with about 20% of patients necessitating over 20,000 IU of factor concentrate annually. Significantly, two-thirds of the cases involved histories of minor trauma that led to the requirement for factor replacement. Only a small fraction, 5%, were identified as positive for inhibitors against factor VIII, with all instances classified under severe hemophilia.

Acknowledging the varied presentations of hemophilia, the pivotal role of transfusion medicine becomes evident in providing essential laboratory services, such as hemostasis and serology testing, screening for inhibitors, and the provision of factor concentrate. This diversity underscores the necessity for a comprehensive management strategy that not only addresses these challenges but also aims to improve patient outcomes. Our study emphasizes the critical importance of timely factor replacement therapy coupled with supportive transfusion medicine practices to effectively mitigate the impact of this inherited coagulation disorder.
